# Genome-wide identification and developmental expression profiling of long noncoding RNAs during *Drosophila* metamorphosis

**DOI:** 10.1038/srep23330

**Published:** 2016-03-21

**Authors:** Bing Chen, Yi Zhang, Xia Zhang, Shili Jia, Shuang Chen, Le Kang

**Affiliations:** 1 State Key Laboratory of Integrated Management of Pest Insects and Rodents, Institute of Zoology, Chinese Academy of Sciences, Beijing 100101, China; 2 Department of Mathematics, Hebei University of Science and Technology/Hebei Laboratory of Pharmaceutical Molecular Chemistry, Shijiazhuang 050018, China; 3 Chengdu Institute of Biology, Chinese Academy of Sciences, Chengdu 610041, China; 4 Beijing Institutes of Life Sciences, Chinese Academy of Sciences, Beijing 100101, China

## Abstract

An increasing number of long noncoding RNAs (lncRNAs) have been discovered with the recent advances in RNA-sequencing technologies. lncRNAs play key roles across diverse biological processes, and are involved in developmental regulation. However, knowledge about how the genome-wide expression of lncRNAs is developmentally regulated is still limited. We here performed a whole-genome identification of lncRNAs followed by a global expression profiling of these lncRNAs during development in *Drosophila melanogaster*. We combined bioinformatic prediction of lncRNAs with stringent filtering of protein-coding transcripts and experimental validation to define a high-confidence set of *Drosophila* lncRNAs. We identified 1,077 lncRNAs in the given transcriptomes that contain 43,967 transcripts; among these, 646 lncRNAs are novel. *In vivo* expression profiling of these lncRNAs in 27 developmental processes revealed that the expression of lncRNAs is highly temporally restricted relative to that of protein-coding genes. Remarkably, 21% and 42% lncRNAs were significantly upregulated at late embryonic and larval stage, the critical time for developmental transition. The results highlight the developmental specificity of lncRNA expression, and reflect the regulatory significance of a large subclass of lncRNAs for the onset of metamorphosis. The systematic annotation and expression analysis of lncRNAs during *Drosophila* development form the foundation for future functional exploration.

The rapid accumulation of data from tilling arrays and high-throughput sequencing indicates that most of a eukaryotic genome is actively transcribed. Many of these transcribed RNAs are untranslatable into proteins and are called noncoding RNAs (ncRNAs). More than half of the mammalian transcriptome is comprised of ncRNAs^1^. These transcripts consist of small ncRNAs (including microRNAs and piRNAs) and long ncRNAs (lncRNA, >200 nucleotides). There is increasing *in vitro* and *in vivo* evidence that lncRNAs play key roles across diverse biological processes^2^,^3^,^4^,^5^, although functions of lncRNAs are largely unknown^6^,^7^. For example, lncRNAs are involved in dosage compensation, genomic imprinting, and regulation of epigenetic marks and gene expression^8^,^9^,^10^. Many lncRNAs are associated with human disease^3^,^10^,^11^,^12^,^13^. Therefore, the discovery and annotation of lncRNAs have attracted increasing attentions in recent years^7^.

Intriguing studies on individual lncRNAs have revealed distinct functions for different lncRNA families. One of the major functions mediated by lncRNAs is related to developmental regulation for pluripotency, differentiation, specialization and homeostasis^3^,^14^,^15^. Several findings that support the biological significance of lncRNAs demonstrate that many lncRNAs exhibit tissue- or cell type-specific expression^16^,^17^,^18^,^19^. Some lncRNAs exhibit expression at specific developmental time point^20^, and thus can be only discovered during narrow developmental time windows. These findings suggest that the complex task involved in developmental regulation requires the fine timing, space, and rate of expression of specific lncRNAs^2^. However, knowledge about how the genome-wide expression of lncRNAs is developmentally regulated is still limited. A systematic profiling of the temporal or spatial expression of lncRNAs during development undoubtedly will facilitate such understanding.

*D. melanogaster* is a cheaper and more amenable genetic model organism for the functional study of lncRNAs compared with mammal models. *Drosophila* also has many benefits for the evolutionary and experimental investigation of lncRNA loci^21^. Many *Drosophila* lncRNAs have been predicted^21^,^22^,^23^,^24^,^25^. Several lncRNAs have been functionally characterized^26^,^27^,^28^, and found to be associated with X inactivation^29^, behaviors^30^, and neuronal disease^31^. However, a systematic identification of lncRNAs from exhaustive transcriptome datasets and global expression profiling of lncRNAs during development processes is still lacking, thus impeding the functional and mechanistic exploration of lncRNAs in *D. melanogaster*.

The annotations of many *Drosophila* transcriptomes from modENCODE ( www.modencode.org) and Flybase ( www.flybase.org) have included expression data at all developmental stages^32^,^33^. These databases provide valuable sources for the systematic catalog of lncRNA genes as well as the developmental profiling of their expression. In this study, we applied integrated methods to discover *D. melanogaster* lncRNAs and verified the predicted lncRNAs with bioinformatic and experimental approaches. The expression profiles of lncRNAs in 27 developmental processes in *D. melanogaster* were analyzed. The results showed high developmental specificity of lncRNA expression and extensive involvement of a large subclass of lncRNAs during *Drosophila* metamorphosis. The systematic annotation and expression profiling of *Drosophila* lncRNAs highlight the fundamental importance of lncRNA enrichment for developmental regulation and lay the foundation for future functional and evolutionary studies.

## Results

### *Drosophila* lncRNA identification and validation

**Figure 1:**
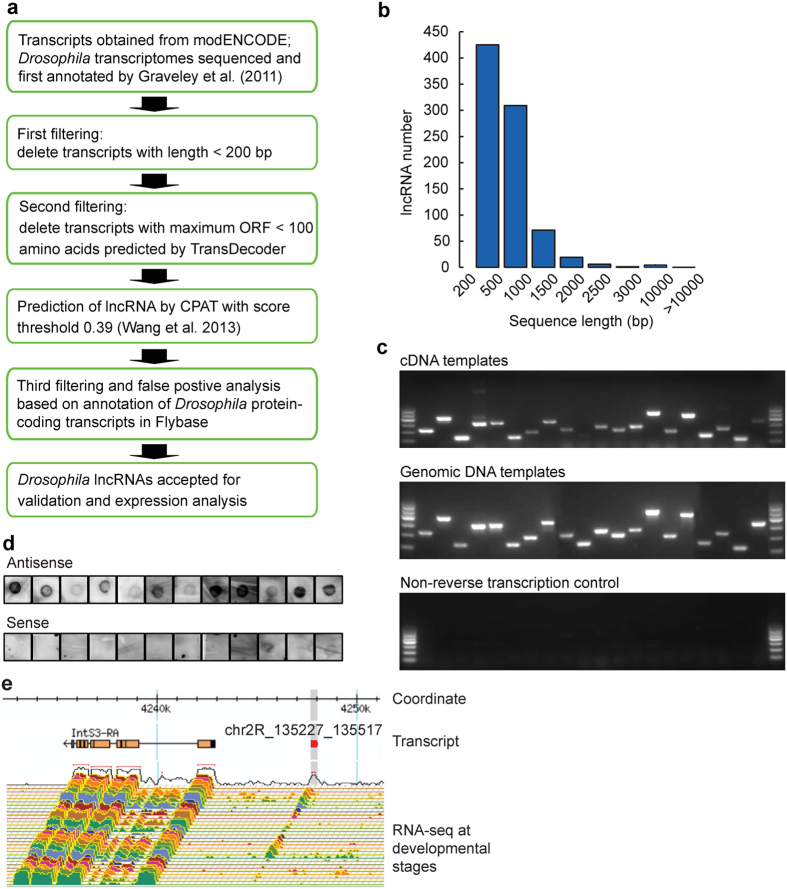
Identification and validation of lncRNAs in *D. melanogaster*. (**a**) Analytical diagram for *Drosophila* lncRNA filtering and prediction. The transcriptome database of *D. melanogaster* contains an annotation of 43,967 transcripts. All transcripts were subject to three-step filterings and a computational prediction of ncRNAs using the program CPAT. False positive rate of lncRNA prediction was evaluated based on protein-coding gene annotations in Flybase. (**b**) Length distribution of the 976 lncRNAs identified in *D. melanogaster*. (**c**) Validation of 20 predicted lncRNAs using RT-PCR. The cDNA transcripts were transcribed from RNA with reverse transcriptase, and then used as PCR templates. PCR templates were genomic DNA in the positive control. In the negative control, RNA samples were subject to the same reverse transcription process with omission of the reverse transcriptase. Lanes on the two sides of gel are 100 DNA ladder. See details about the 20 lncRNAs in Supplementary Table S1 and Supplementary data 1. (**d**) Validation of 12 predicted intergenic lncRNAs by RNA dot blot. The 12 lncRNAs are among the 20 lncRNAs and had been validated by RT-PCR. Total 10 μg RNA were hybridized with Biotin labelled probes on nylon membrane. The 12 lncRNAs are as follows: chr3L_18316644_18317098, chr2L_18795816_18796184, chr3LHet_2236180_2236548, chr2L_21798044_21799854, chr3RHet_1825840_1826355, MIP19078, chrX_3968951_3969506, chr3L_18686588_18686941, chr2L_3268204_3268702, chr2L_8887436_8888049, hr2L_22705365_22706170, chr2R_135227_135517 (also see Supplementary Table S1). (**e**) Example of a validated novel lncRNA candidate, i.e., chr2R_135227_135517. The chromosome coordinate based on Flybase version FB2014_06 is shown within a range of approximately 30 kilo-bp. The lncRNA indicated by red arrow neighbours coding gene *IntS3*. Bottom colourful tracks show the expression of transcripts from RNA-seq reads in 27 developmental stage, from 0–2-h embryos to 30-d adults.

We identified putative lncRNAs from *D. melanogaster* transcriptomes deposited in modENCODE. The database contains 43,967 transcripts from both poly(A) and total RNAs that are expressed at 27 distinct developmental stages from embryo to adult^32^. We first filtered the transcriptomes of transcripts that are shorter than 200 bp in total length or predicted to harbor maximum open reading frames (ORFs) encoding longer than 100 amino acids (Fig. 1a). Protein coding potential of transcripts was evaluated by TransDecoder ( http://transdecoder.github.io). We then used the software CPAT to predict lncRNA, which is expected to yield more than 96% sensitivity and specificity^34^. As a result, we identified 1,077 putative lncRNAs (>200 base pair, or bp) in *D. melanogaster*. We last evaluated the false positive rate of prediction based on protein annotation in Flybase ( http://flybase.org/). Blast against the *Drosophila* protein-coding transcript sequences (CDS) showed that 9% of the putative lncRNAs, i.e., 101 transcripts, were annotated as CDS in Flybase, even though the coding proteins of most (62%) of the CDS have not been reported. Therefore, only the remaining 976 lncRNAs were accepted and used for the following examination (Supplementary data 1). The predicted lncRNAs are distinct from CDS in coding probability (Supplementary Fig. S1). Among these lncRNAs, 330 transcripts have been annotated as lncRNAs in modENCODE, Flybase, or previous publications^21^,^23^ consisting of 34% of all lncRNAs predicted. The 646 *Drosophila* lncRNAs are therefore putatively novel.

The predicted lncRNAs had an average length of 621 bp. Most (87%) of the lncRNAs were less than 1,000 bp long (Fig. 1b and Supplementary data 1). The averaged size of CDS is 11,254 bp. The size of lncRNAs is much shorter than that of CDS. The averaged size of the 330 lncRNAs is 3,490 bp, and that of the 646 novel lncRNAs is 1,099 bp (Supplementary data 1). The 330 lncRNAs are longer than the 646 lncRNAs mainly because a large proportion (47%) of the novel lncRNAs range between 200–500 bp; In contrast, 39% of the annotated lncRNAs range between 500–1,000 bp, and 19% are longer than 3,000 bp (Supplementary Fig. S2a). The CDS have 6.1 exons in average. The 646 lncRNAs have 1.4 exons while the 330 lncRNAs have 2.2 exons (Supplementary data 1). The averaged size of introns of CDS is 8,270 bp. The size of introns of the 330 lncRNAs is 2,910 bp, and that of the 646 lncRNAs is 501 bp (Supplementary Fig. S2b). The results indicate that we have identified many previously annotated lncRNAs as well as a subset of novel lncRNAs that are distinct in gene structure in *D. melanogaster*.

To verify that these lncRNAs were transcribed RNA but not artificial products or amplified from genomic DNA or contaminants, 20 lncRNAs, including 12 intergenic lncRNAs, were randomly selected from the novel lncRNAs and experimentally validated by RT-PCR. Among the 20 lncRNAs tested, 19 yielded PCR products of the right size and correct sequence from their cDNA templates (Fig. 1c). The 12 intergenic lncRNAs were randomly selected from the 19 lncRNAs and further tested by RNA dot hybridization. Eleven lncRNAs exhibited clear RNA blot with antisense probe but not with sense probe, i.e., the negative control (Fig. 1d). The genomic structures of the 12 lncRNAs were annotated based on Flybase (Supplementary Fig. S3). Detailed analysis on one intergenic lncRNA, i.e., chr2R_135227_135517, revealed its prominent expression in many developmental stages, even though the expression level was low compared with that of the neighboring coding genes (Fig. 1e).

### Developmental specificity of lncRNA expression in *D. melanogaster*

**Figure 2:**
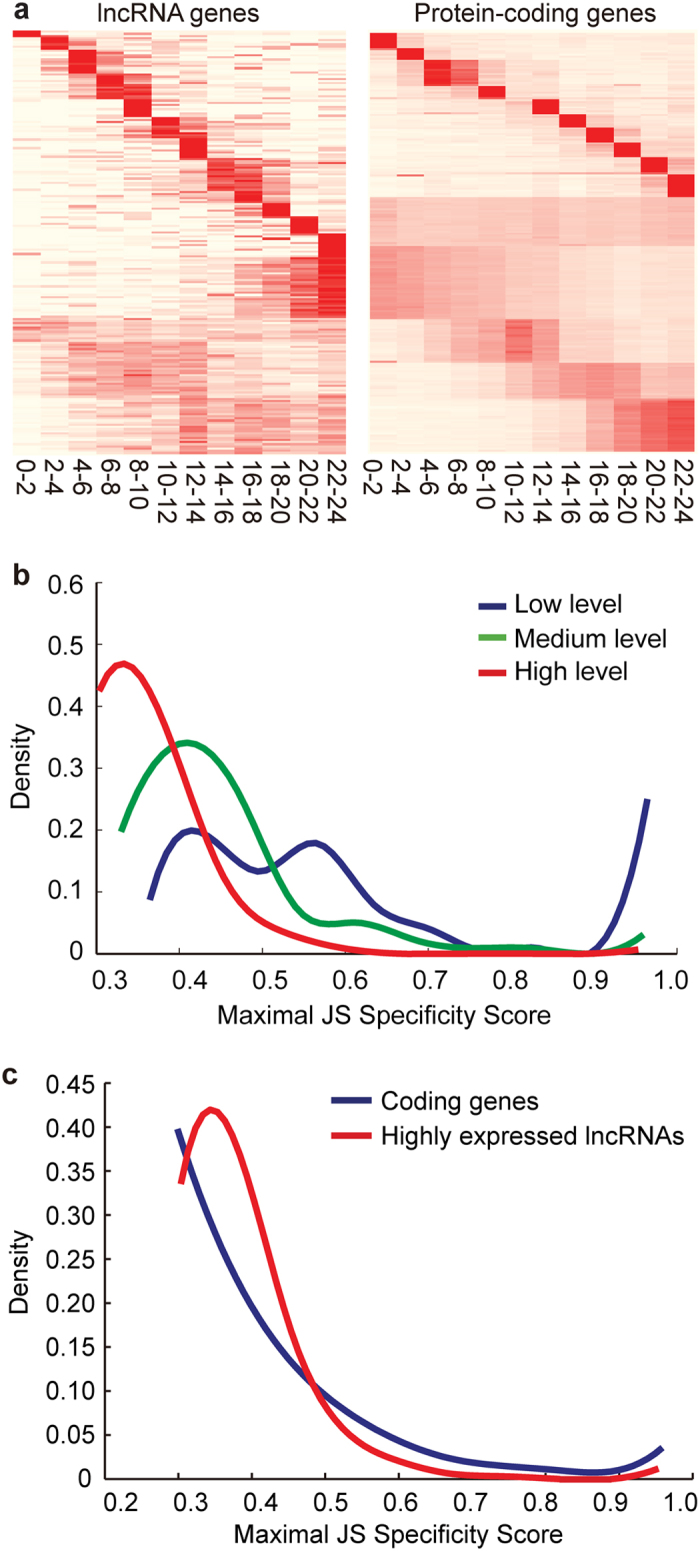
Developmental specificity of the expression of lncRNAs and coding genes at the embryonic stages in *D. melanogaster*. (**a**) Heatmap of the gene expression of lncRNAs and coding gene loci (rows) across 12 embryonic stages (columns). The 976 lncRNAs (left) and 33,074 protein-coding RNAs (right) were clustered separately based on normalized expression values using *k*-means (*k* = 10) with a city-block distance matrix. Embryos were collected at 2 h intervals during the 24 h developmental period. (**b**) Developmental expression specificity of lncRNAs at different levels of expression. Shown are the distributions of Shannon entropy-based temporal specificity scores that were calculated for the three groups of lncRNA loci, i.e., the highly, moderately, and lowly expressed lncRNAs. These lncRNAs were grouped based on their expression levels averaged across the embryonic stages. (**c**) Developmental specificity of the expression of coding genes and highly expressed lncRNAs. The sliding window width is 0.1.

We examined the possible functions of the 976 lncRNAs in developmental regulation by analyzing their developmental profiling and specificity of expression at different stages. We investigated the pattern of gene expression changes with embryonic development at 0 h to 24 h and then compared the expression profiles between lncRNAs and protein-coding loci (Fig. 2a). Some lncRNAs were highly expressed in one of the 12 time points of embryonic development. For example, 133 lncRNAs consisting of 20.7% of the total expressed lncRNAs were specifically highly expressed at the 22 h to 24 h embryonic stage, the critical time point close to the metamorphosis from embryo to larva^35^. This percentage of lncRNAs was significantly higher than that (5.2%) of protein genes highly expressed at this specific stage (Fisher’s exact test, *P* < 3.6 × 10^−15^). The majority (69%) of expressed lncRNAs with 454 transcripts demonstrated a more developmental stage-specific expression, relative to 39% of the protein-coding RNAs (Fisher’s exact test, *P* < 3.4 × 10^*−*14^) (Fig. 2a). The exclusion of 941 housekeeping gene transcripts (Supplementary data 2) from CDS didn’t alter the proportion (i.e., 38.6%) of CDS exhibiting developmental-specific high expression (Supplementary Fig. S4).

To further test whether or not lncRNAs have a more temporally restricted expression than coding genes, we introduced a measure of expression level divergence over developmental time, i.e., a Shannon entropy-based specificity score per locus (JS)^20^. The JS value ranged from 0 to 1, and the expression pattern with JS = 1 indicated perfect specificity. To rule out that the different specificities resulted from the low expression level of lncRNAs, we compared the specificity values of lncRNAs at different expression levels. The developmental specificities of lncRNA expression at different levels were compared in two ranges of maximal JS specificity score. The JS difference between lowly and moderately expressed lncRNAs was significant at JS < 0.9 (Mann–Whitney *U* Test, *P* < 2.3 × 10^−12^). The difference between moderately and highly expressed lncRNAs was also significant (*P* < 9.6 × 10^−15^). The proportion of lncRNAs with extreme specificity, with JS in the range of 0.9 to 1.0, was also compared. Approximately 26.5% lowly expressed lncRNAs, 3.8% moderately expressed lncRNAs, and 1.1% highly expressed lncRNAs exhibited extreme temporal-restricted expression. A significant difference in the proportion was detected between lowly and moderately expressed lncRNAs (Fisher’s exact test, *P* < 6.5 × 10^−12^) but not between moderately and highly expressed lncRNAs (*P = *0.071) (Fig. 2b). The results suggest that the lowly expressed lncRNAs significantly contribute to the extreme JS value. Therefore, to compare the expression specificities of lncRNAs and coding RNAs, we excluded the lowly expressed lncRNA, and only compared the coding RNAs and 331 highly expressed lncRNAs in embryos. A significant difference in developmental specificity was observed between the coding genes and highly expressed lncRNAs (Mann-Whitney *U* Test, *P* = 0.039) (Fig. 2c).

**Figure 3:**
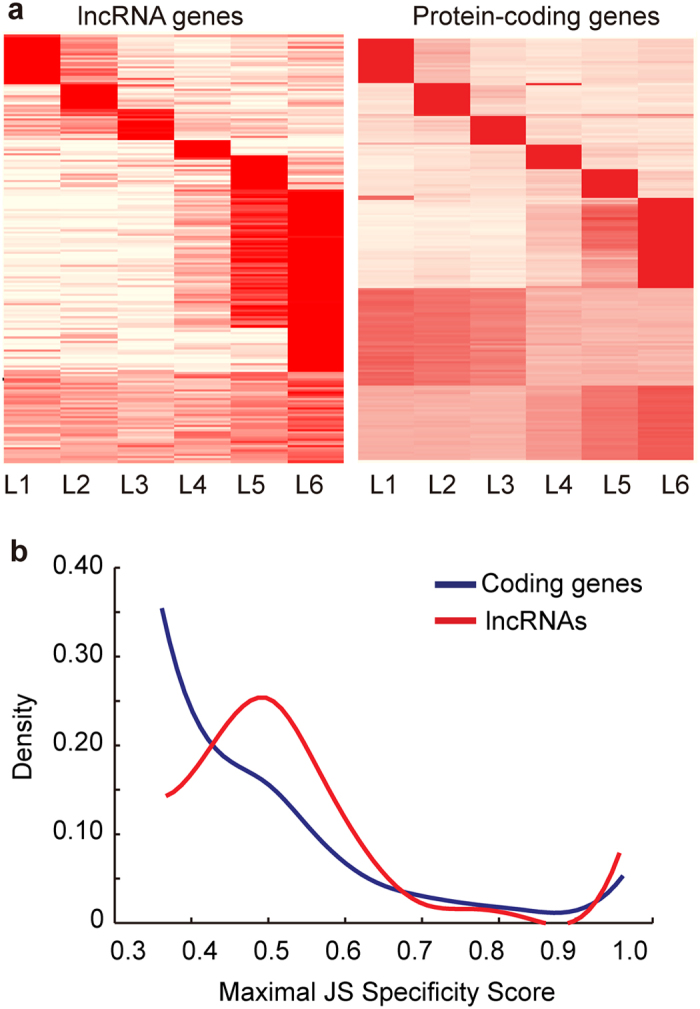
Developmental expression of lncRNAs and coding genes at larval stages in *D. melanogaster.* (**a**) Heatmap of gene expression of lncRNAs and coding gene loci (rows) across 6 larval stages (columns). The 976 lncRNAs (left) and 33,074 protein-coding RNAs (right) were clustered separately based on normalized expression values using k-means (*k* = 8) with a city-block distance matrix. The larval stage is divided into six sub-stages, i.e., L1 larvae, L2 larvae, L3 larvae (12 hour post-moult), L4 larvae at puff stage 1 with dark blue gut marked with 0.05% bromophenol blue, L5 larvae at puff stage 2–6 with light blue gut, and L6 larvae at puff stage 7–9 with clear gut. (**b**) Developmental expression specificity of coding genes and highly expressed lncRNAs. Shown are the distributions of Shannon entropy-based temporal specificity scores. The sliding window width is 0.1.

The developmental specificity of lncRNA expression was also examined at the larval stage (Fig. 3). The majority (79%) of the lncRNAs had high stage-specific expression. By contrast, half (50%) coding genes were specifically highly expressed (*P* < 3.9 × 10^−14^). The percentage of highly expressed genes (15,585 out of 31,212) remained unchanged after the housekeeping genes were excluded for the analysis (Supplementary Fig. S4). Approximately 42% lncRNAs were specifically highly expressed at the last larval stage, the critical time for the transformation from larval to prepupal stage (Fig. 3a). The maximal JS specificity of the coding genes was obtained at 0.36, whereas that of the lncRNAs was obtained at 0.5. Up to 11% of the lncRNAs reached an extreme specificity, which was significantly higher than that (4%) of the protein-coding genes (*P* < 4.1 × 10^−11^) (Fig. 3b). The developmental specificity of the lncRNA expression observed at the pupal stage was not as prominent as that at the embryonic and larval stages (Supplementary Fig. S5). No significant difference in the maximum JS specificity was detected between the lncRNAs and coding RNAs (Supplementary Fig. S5).

## Discussion

An increasing number of ncRNAs have been discovered with the deeper-sequencing of transcriptomes and the accumulation of transcript data. Thousands of ncRNA loci (both small and long RNAs) have been suggested to exist in the *D. melanogaster* genome^21^,^22^,^25^,^36^. In the present study, we combined high-accuracy prediction of lncRNAs with a stringent filtering of putative protein-coding transcripts to define a high-confidence set of lncRNAs. As lncRNAs typically present a cell type-specific expression as well as a lower expression than protein-coding genes, we searched lncRNAs in a rich source of transcriptomes that are originated from 27 developmental stages. We identified 1,077 lncRNAs from 43,967 transcripts, among which 646 lncRNAs have not been previously reported. These lncRNAs were missed from previous annotation mainly because different methods for ncRNA annotation had been used. For example, we used CPAT program for ncRNA prediction, which relies on a logistic regression model built with sequence features^34^. Many novel lncRNAs are short and located in intergenic region. However, previous methods identified ncRNA mainly based on the annotation of gene structure/gene model in flybase^32^. Experimental validations further confirm the reliability of the identified lncRNAs. These lncRNAs provide a high-quality resource of lncRNAs for future functional and evolutionary studies. Nevertheless, more lncRNAs in *D. melanogaster* are expected to be discovered with deeper-sequencing in different tissues and in individuals exposed to different environmental conditions.

Analysis of the developmental *in vivo* expression profile of *Drosophila* lncRNAs highlighted the high developmental specificity of lncRNA expression. The expression profiling of the 976 lncRNAs showed that lncRNAs were expressed in a much narrower time window than protein-coding genes. Thus, the former was more highly temporally restricted than the latter. The developmental specificity of expression was demonstrated at the embryonic, larval, and pupal stages examined. Recent results prove that lncRNAs have important functions in cell fate decision, differentiation, migration and signaling^6^,^18^,^20^,^37^,^38^. Our findings suggest that lncRNAs could be involved in regulation of the timing of developmental transition.

An interesting finding revealed in the study is that a large subclass of lncRNAs are prominently upregulated at the developmental stage of metamorphosis. Twenty-one percent of lncRNAs were highly upregulated at 22–24 h of embryos (Fig. 2) and 42% of lncRNAs were greatly upregulated at the puff stage 7–9 (i.e., L6) of larvae (Fig. 3). The massive upregulation of lncRNAs coincides with the onset of metamorphosis from embryos to larvae and from larvae to prepupae. A similar phenomenon was reported in the developmental expression of lncRNAs in zebrafish^20^. The results indicate that lncRNA enrichment in development could be important for the transformation and organogenesis in animals, even though further experiments are needed to demonstrate it.

Numerous studies have provided evidence that lncRNAs are crucial players in cell differentiation and development^6^. For example, lncRNA *Fendrr* presents lateral mesoderm-specific expression which is critical for proper wall development^18^. LncRNAs also exhibit conserved functions in animal embryonic development^2^,^15^,^39^. Some lncRNAs are involved in processes directing embryonic stem cell pluripotency and differentiation^15^. Interestingly, expression of many lncRNAs is regulated in response to hormone^40^. The coordinated high expression of numerous lncRNAs in late embryos and larvae are highly in synchrony with the high titer pulses of the steroid hormone ecdysone in *Drosophila*^40^,^41^. Temporal boundaries in the *Drosophila* life cycle is defined by ecdysone, whose titrate pulses trigger the major postembryonic developmental transitions, including molting and metamorphosis^35^,^41^. Ecdysone exerts its effects through a regulatory cascade of ecdysone - ecdysone receptors (e.g., EcR and USP) - target genes. Some lncRNAs act as coactivator^42^ or repressor^43^ of hormone receptors and respond to hormone titration^5^. The upregulation of lncRNAs could be responding to a temporal signal that occurs in association with or in parallel with the ecdysone - EcR signaling pathway. Therefore, lncRNAs may mediate the timing of embryo-to-pupae cell fate decisions by interacting with ecdysone and temporal protein regulators of differentiation.

## Methods

### *D. melanogaster* transcriptomes and transcript expression

The transcriptome data of *D. melanogaster* were obtained from modENCODE ( http://www.modencode.org). The transcriptomes have an annotation of 43,967 transcripts that are pooled from RNA expressed at 27 distinct developmental stages from embryo to adult. These stages include 12 embryonic stages divided at 2 h intervals for 24 h, 6 larval stages, 6 pupal stages, and 3 male and 3 female adult stages. The transcriptomes of each stage were generated from poly(A) RNAs, but embryonic transcriptomes were generated from both poly(A) RNAs and total RNAs^32^. A variety of methods and sequencing data, such as complementary tiling microarray and RNA-Seq, long read sequencing, and genome mapping, were used for the prediction of novel splice junctions and the mapping and annotation of transcripts and genes. RNAs from three biological replicates of each sample were prepared for tiling arrays^32^. Here ‘transcript’ refers to a RNA molecule that transcribes from a genomic loci whereas ‘gene’ refers to one or more transcripts that share exons. The expression data from poly(A) RNAs were used in this study.

### *Drosophila* lncRNA identification

*Drosophila* lncRNAs were predicted from the 43,967 transcripts. Open reading frames (ORFs) in the transcripts were evaluated using the program TransDecoder ( http://transdecoder.github.io). Transcripts possessing a maximum ORF encoding >100 amino acids were filtered. A state-of-the-art and popular software CPAT^34^ was used to predict ncRNAs in the transcripts. CPAT is an alignment-free method and uses a logistic regression model built with four sequence features including ORF size, ORF coverage, Fickett TESTCODE statistic and hexamer usage bias. CPAT has been optimized for prediction of *Drosophila* lncRNA with coding probability cutoff at 0.39 (i.e., coding probability <0.39 indicates ncRNA, also see: http://rna-cpat.sourceforge.net/)^34^. Prediction with CPAT yields a high sensitivity (0.96) and high specificity (0.97) in theory^34^. The predicted transcripts were finally blasted against CDS annotated in Flybase (built 5.54) to evaluate false positive rate of the prediction. The ncRNAs that were filtered of all CDS and longer than 200 bp genes were labeled as putative *Drosophila* lncRNAs for further experimental validation and expression analysis.

### Validation of *Drosophila* lncRNAs by RT-PCR and dot-blot hybridization

Twenty intergenic lncRNAs that were randomly selected from the predicted lncRNAs were validated by RT-PCR and RNA dot hybridization (Supplementary Table S1). Total RNA was isolated from whole body tissues of 5-d-old female adults of *D. melanogaster* (strain *OreR*) and was treated with Dnase I. Reverse transcription was then performed using Oligo-d(T) following the Omniscript RT kit instruction (QIAGEN, Cat. no. 205110). We first used RT-PCR to validate the candidate lncRNAs. The amplification conditions were as follows: 94 °C for 1 min and 40 cycles of 94 °C for 30 s, 58 °C for 30 s, and 72 °C for 30 s. Thirty cycles of amplification were used for the amplification. The RT-PCR products were purified and were sent for direct sequencing to verify their sequences. Positive and negative controls were also subjected to RT-PCR. The positive control contained genomic DNA as the template for PCR to test the validity of the designed primers and the PCR system used. The negative control had no reverse transcriptase added for reverse transcription. Expression of 12 lncRNAs were further validated by RNA hybridization. These lncRNAs except one, i.e. chr2L_21798044_21799854, are single exoned. High specific RNA probes were first synthesized using Biotin RNA labeling mix (Roche, Cat. no. 11685597910) and T7 RNA polymerase (Promega, Cat. no. p2075). Total 10 μg RNA were hybridized with the probes on BrightStar-Plus nylon membrane (Ambion, Cat. no. AM10102). After blocking and wash with Chemiluminescent Nucleic Acid detection Module (Thermal Scientific, Cat. no. 89880), the biotin-labeled RNA was detected using CCD imaging station system (Carestream Health, USA). Primers for hybridization probes were the same as those used for the RT-PCR. Details on the selected ncRNAs and PCR primers can be found in Supplementary Table S1. Note that all these primers were designed to amplify exonic region.

### Developmental specificity of lncRNA expression

FPKM expression levels were normalized to obtain the relative expression levels over the course of development and then to visualize developmental expression profiles via heatmaps. The lncRNAs and protein-coding loci were separately clustered using Cluster 3.0 software using k-means with a city-block distance matrix^44^. The transcripts were clustered into 10 groups at the embryonic stage, 8 groups at the larval stage, and 9 groups at the pupal stage to achieve distinct clusters. The 976 lncRNAs identified in this study and the 33,074 protein-coding transcripts were analyzed for the heatmap and developmental specificity of expression. The protein-coding transcripts were further filtered to exclude housekeeping genes for the analysis of developmental specificity. By comparing with human housekeeping genes^45^, a total of 941 orthologous transcripts from 172 genes were taken as *Drosophila* housekeeping genes (Supplemental data 2).

The developmental specificity of the expression of a transcript was evaluated following the measure used by Trapnell *et al.*^46^ and Cabili *et al.*^16^. The entropy-based specificity measure quantifies the similarity between the expression pattern of an ncRNA or coding transcript and another predefined transcript expressed at only one stage. The developmental specificity is defined as the maximal specificity score of the transcript expression pattern across all developmental stages, i.e., maximal JS specificity score. In the present study, we examined the specificity measures of transcripts expressed at the embryonic, larval, and pupal stages.

### Statistical analyses

Fisher’s exact test was performed to compare the difference in the number of genes. All lncRNAs were sorted based on their averaged expression level in different periods of a developmental stage, and categorized according to their ranking of expression. The difference in JS values between gene sets was analyzed by Mann-Whitney *U* Test.

## Additional Information

**How to cite this article**: Chen, B. *et al.* Genome-wide identification and developmental expression profiling of long noncoding RNAs during *Drosophila* metamorphosis. *Sci. Rep.* **6**, 23330; doi: 10.1038/srep23330 (2016).

## Supplementary Material

Supplementary Information

Supplementary Data 1

Supplementary Data 2

## References

[b1] Carninci P. & Hayashizaki Y. Noncoding RNA transcription beyond annotated genes. Curr Opin Genet Dev 17, 139–144, doi: 10.1016/j.gde.2007.02.008 (2007). 17317145

[b2] Pauli A., Rinn J. L. & Schier A. F. Non-coding RNAs as regulators of embryogenesis. Nat Rev Genet 12, 136–149, doi: 10.1038/nrg2904 (2011). 21245830 PMC4081495

[b3] Batista P. J. & Chang H. Y. Long noncoding RNAs: cellular address codes in development and disease. Cell 152, 1298–1307, doi: 10.1016/j.cell.2013.02.012 (2013). 23498938 PMC3651923

[b4] Pandey R. R. & Kanduri C. Transcriptional and posttranscriptional programming by long noncoding RNAs. Prog Mol Subcell Biol 51, 1–27, doi: 10.1007/978-3-642-16502-3_1 (2011). 21287131

[b5] Wang K. & Chang H. Molecular Mechanisms of long noncoding RNAs. Mol cell 43, 904–914 (2011). 21925379 10.1016/j.molcel.2011.08.018 PMC3199020

[b6] Fatica A. & Bozzoni I. Long non-coding RNAs: new players in cell differentiation and development. Nat Rev Genet 15, 7–21, doi: 10.1038/nrg3606 (2014). 24296535

[b7] Mattick J. S. & Rinn J. L. Discovery and annotation of long noncoding RNAs. Nat Struct Mol Biol 22, 5–7, doi: 10.1038/nsmb.2942 (2015). 25565026

[b8] Guttman M. & Rinn J. L. Modular regulatory principles of large non-coding RNAs. Nature 482, 339–346, doi: 10.1038/nature10887 (2012). 22337053 PMC4197003

[b9] Heo J. B. & Sung S. Vernalization-mediated epigenetic silencing by a long intronic noncoding RNA. Science 331, 76–79, doi: 10.1126/science.1197349 (2011). 21127216

[b10] Lee J. T. & Bartolomei M. S. X-inactivation, imprinting, and long noncoding RNAs in health and disease. Cell 152, 1308–1323, doi: 10.1016/j.cell.2013.02.016 (2013). 23498939

[b11] Esteller M. Non-coding RNAs in human disease. Nat Rev Genet 12, 861–874, doi: 10.1038/nrg3074 (2011). 22094949

[b12] Ng S. Y., Lin L., Soh B. S. & Stanton L. W. Long noncoding RNAs in development and disease of the central nervous system. Trends Genet, doi: 10.1016/j.tig.2013.03.002 (2013). 23562612

[b13] Qureshi I. A. & Mehler M. F. Emerging roles of non-coding RNAs in brain evolution, development, plasticity and disease. Nat Rev Neurosci 13, 528–541 (2012). 22814587 10.1038/nrn3234 PMC3478095

[b14] Guttman M. *et al.* lincRNAs act in the circuitry controlling pluripotency and differentiation. Nature 477, 295–300, doi: 10.1038/nature10398 (2011). 21874018 PMC3175327

[b15] Dinger M. E. *et al.* Long noncoding RNAs in mouse embryonic stem cell pluripotency and differentiation. Genome Res 18, 1433–1445, doi: 10.1101/gr.078378.108 (2008). 18562676 PMC2527704

[b16] Cabili M. N. *et al.* Integrative annotation of human large intergenic noncoding RNAs reveals global properties and specific subclasses. Genes Dev 25, 1915–1927, doi: 10.1101/gad.17446611 (2011). 21890647 PMC3185964

[b17] Mercer T. R., Dinger M. E., Sunkin S. M., Mehler M. F. & Mattick J. S. Specific expression of long noncoding RNAs in the mouse brain. Proc Natl Acad Sci USA 105, 716–721, doi: 10.1073/pnas.0706729105 (2008). 18184812 PMC2206602

[b18] Grote P. *et al.* The tissue-specific lncRNA Fendrr is an essential regulator of heart and body wall development in the mouse. Dev Cell 24, 206–214, doi: 10.1016/j.devcel.2012.12.012 (2013). 23369715 PMC4149175

[b19] Tsoi L. C. *et al.* Analysis of long non-coding RNAs highlights tissue-specific expression patterns and epigenetic profiles in normal and psoriatic skin. Genome Biol, 16, 24, doi: 10.1186/s13059-014-0570-4 (2015). 25723451 PMC4311508

[b20] Pauli A. *et al.* Systematic identification of long noncoding RNAs expressed during zebrafish embryogenesis. Genome Res 22, 577–591, doi: 10.1101/gr.133009.111 (2012). 22110045 PMC3290793

[b21] Young R. S. *et al.* Identification and properties of 1,119 candidate lincRNA loci in the *Drosophila melanogaster* genome. Genome Biol Evol 4, 427–442, doi: 10.1093/gbe/evs020 (2012). 22403033 PMC3342871

[b22] Bradley R. K. *et al.* Evolutionary modeling and prediction of non-coding RNAs in *Drosophila*. PLoS One 4, e6478, doi: 10.1371/journal.pone.0006478 (2009). 19668382 PMC2721679

[b23] Hiller M. *et al.* Conserved introns reveal novel transcripts in *Drosophila melanogaster*. Genome Res 19, 1289–1300, doi: 10.1101/gr.090050.108 (2009). 19458021 PMC2704441

[b24] Inagaki S. *et al.* Identification and expression analysis of putative mRNA-like non-coding RNA in *Drosophila*. Genes Cells 10, 1163–1173 (2005). 16324153 10.1111/j.1365-2443.2005.00910.x

[b25] Tupy J. L. *et al.* Identification of putative noncoding polyadenylated transcripts in *Drosophila melanogaster*. Proc Natl Acad Sci USA 102, 5495–5500, doi: 10.1073/pnas.0501422102 (2005). 15809421 PMC555963

[b26] Sanchez-Elsner T. Noncoding RNAs of trithorax response elements recruit *Drosophila* Ash1 to Ultrabithorax. Science 311, 1118–1123, doi: 10.1126/science.1117705 (2006). 16497925

[b27] Soshnev A. A. *et al.* A conserved long noncoding RNA affects sleep behavior in *Drosophila*. Genetics 189, 455–468, doi: 10.1534/genetics.111.131706 (2011). 21775470 PMC3189806

[b28] Prasanth K., Rajendra T., Lal A. & Lakhotia S. Omega speckles-a novel class of nuclear speckles containing hnRNPs associated with noncoding hsr-omega RNA in *Drosophila*. J Cell Sci 113, 3485–3497 (2000). 10984439 10.1242/jcs.113.19.3485

[b29] Lim C. K. & Kelley R. L. Autoregulation of the Drosophila noncoding roX1 RNA gene. PLoS Genet 8, e1002564, doi: 10.1371/journal.pgen.1002564 (2012). 22438819 PMC3305356

[b30] Li M. *et al.* The novel long non-coding RNA CRG regulates *Drosophila* locomotor behavior. Nucleic Acids Res 40, 11714–11727, doi: 10.1093/nar/gks943 (2012). 23074190 PMC3526303

[b31] Mutsuddi M., Marshall C. M., Benzow K. A., Koob M. D. & Rebay I. The spinocerebellar ataxia 8 noncoding RNA causes neurodegeneration and associates with staufen in *Drosophila*. Curr Biol 14, 302–308 (2004). 14972680 10.1016/j.cub.2004.01.034

[b32] Graveley B. R. *et al.* The developmental transcriptome of *Drosophila melanogaster*. Nature 471, 473–479, doi: 10.1038/nature09715 (2011). 21179090 PMC3075879

[b33] Roy S. *et al.* Identification of functional elements and regulatory circuits by *Drosophila* modENCODE. Science 330, 1787–1797 (2010). 21177974 10.1126/science.1198374 PMC3192495

[b34] Wang L. *et al.* CPAT: Coding-Potential Assessment Tool using an alignment-free logistic regression model. Nucleic Acids Res 41, e74, doi: 10.1093/nar/gkt006 (2013). 23335781 PMC3616698

[b35] Bashirullah A. *et al.* Coordinate regulation of small temporal RNAs at the onset of *Drosophila* metamorphosis. Dev Biol 259, 1–8, doi: 10.1016/s0012-1606(03)00063-0 (2003). 12812783

[b36] Li Z. *et al.* Detection of intergenic non-coding RNAs expressed in the main developmental stages in Drosophila melanogaster. Nucleic Acids Res 37, 4308–4314, doi: 10.1093/nar/gkp334 (2009). 19451167 PMC2715228

[b37] Cesana M. *et al.* A long noncoding RNA controls muscle differentiation by functioning as a competing endogenous RNA. Cell 147, 358–369, doi: 10.1016/j.cell.2011.09.028 (2011). 22000014 PMC3234495

[b38] Ng S. Y., Johnson R. & Stanton L. W. Human long non-coding RNAs promote pluripotency and neuronal differentiation by association with chromatin modifiers and transcription factors. EMBO J 31, 522–533, doi: 10.1038/emboj.2011.459 (2012). 22193719 PMC3273385

[b39] Ulitsky I., Shkumatava A., Jan C. H., Sive H. & Bartel D. P. Conserved function of lincRNAs in vertebrate embryonic development despite rapid sequence evolution. Cell 147, 1537–1550, doi: 10.1016/j.cell.2011.11.055 (2011). 22196729 PMC3376356

[b40] Knoll M., Lodish H. F. & Sun L. Long non-coding RNAs as regulators of the endocrine system. Nature reviews. Endocrinology 11, 151–160, doi: 10.1038/nrendo.2014.229 (2015). PMC4376378 25560704

[b41] Riddiford L. M., Cherbas P. & Truman J. W. Ecdysone receptors and their biological actions. Vitam Horm 60, 1–73 (2000). 11037621 10.1016/s0083-6729(00)60016-x

[b42] Lee J. T. Epigenetic regulation by long noncoding RNAs. Science 338, 1435–1439, doi: 10.1126/science.1231776 (2012). 23239728

[b43] Kino T., Hurt D. E., Ichijo T., Nader N. & Chrousos G. P. Noncoding RNA Gas5 is a growth arrest and starvation-associated repressor of the glucocorticoid receptor. Sci Signal 3, ra8 (2010). 20124551 10.1126/scisignal.2000568 PMC2819218

[b44] de Hoon M. J., Imoto S., Nolan J. & Miyano S. Open source clustering software. Bioinformatics 20, 1453–1454, doi: 10.1093/bioinformatics/bth078 (2004). 14871861

[b45] Eisenberg E. & Levanon E. Y. Human housekeeping genes, revisited. Trends Genet 29, 569–574, doi: http://dx.doi.org/10.1016/j.tig.2013.05.010 (2013). 23810203 10.1016/j.tig.2013.05.010

[b46] Trapnell C. *et al.* Transcript assembly and quantification by RNA-Seq reveals unannotated transcripts and isoform switching during cell differentiation. Nat Biotechnol 28, 511–515, doi: 10.1038/nbt.1621 (2010). 20436464 PMC3146043

