# Supplementary material for: Genome-wide identification and developmental expression profiling of long noncoding RNAs during Drosophila metamorphosis
Source: Sci Rep. 2016 Mar 21;6:23330. doi: 10.1038/srep23330 (PMC4800424; doi:10.1038/srep23330)
Supplement: Supplementary Information [file srep23330-s1.doc]

Supplementary Information

**Genome-wide identification and developmental expression profiling of long noncoding RNAs during *Drosophila* metamorphosis**

Bing Chen, Yi Zhang, Xia Zhang, Shili Jia, Shuang Chen, Le Kang

# Supplementary Table S1. PCR primers used to amplify the 20 lncRNAs identified in *D. melanogaster*. These lncRNAs were randomly selected for experimental validation from the novel 646 lncRNAs identified in *D. melanogaster*.

| Number | lncRNA name | Primer pair | Primer sequence | Amplicon size (bp) |
| --- | --- | --- | --- | --- |
| 1 | chr2R_7592327_7592857 | nc1F | TGTTGCAGTCACGAGTTTGG | 229 |
|  |  | nc1R | AATTAATTTGTGACGCGCGC |  |
| 2 | chr3L_17509183_17510009 | nc2F | TGACACACTCCATCACTGCT | 414 |
|  |  | nc2R | CGAGAACCCTAGAGAGCCTG |  |
| 3 | chr3R_2645852_2646154 | nc3F | CCCTCCTTAGCCAACCTGAA | 120 |
|  |  | nc3R | CCTGAGGCTGGGATCTACTG |  |
| 4 | chr3L_13074711_13075073 | nc4F | GTGCAATTGGGCCTTGACTT | 307 |
|  |  | nc4R | CGCTGTCAGTTTTCACACCA |  |
| 5 | MIP19078 | nc5F | GATGTGCTCGGATTATGGCG | 314 |
|  |  | nc5R | TAGGGGCATCAAGTTCGGTT |  |
| 6 | chr3LHet_2236180_2236548 | nc6F | TCGATGTAATTGCTTGTTTTGGC | 127 |
|  |  | nc6R | ACAAGGAGTCACGTTAATCCAAC |  |
| 7 | chr2R_135227_135517 | nc7F | GCAAGTGTCGCAATATCCGT | 197 |
|  |  | nc7R | GGCTTATCTACCCGGCTTGA |  |
| 8 | chr3R_9991708_9992662 | nc8F | CAGAATTCCCATGGCTGAGT | 336 |
|  |  | nc8R | TAGGTCGGTCGGAAATAACG |  |
| 9 | chr2L_3268204_3268702 | nc9F | TTAGGTCAGAAGCCGTCGTT | 229 |
|  |  | nc9R | CGCCTGTTTGTGGTTATGTG |  |
| 10 | chr2L_11663230_11663806 | nc10F | GACTCGATCGGGGAAAAGGA | 162 |
|  |  | nc10R | TGAAGAACTACTGCCACCCA |  |
| 11 | chr2L_22705365_22706170 | nc11F | CAGCACGAAGATCCAACAGA | 263 |
|  |  | nc11R | CATCTGCCCGAGATCGTATT |  |
| 12 | chr2L_18795816_18796184 | nc12F | CACACACACATTCCCACACA | 219 |
|  |  | nc12R | GAGCGATGACAGACGAATGA |  |
| 13 | chrX_6485101_6485457 | nc13F | TGGCAGAGAAAGGAAAACCG | 246 |
|  |  | nc13R | GTGCCGCGAGTACCAAAA |  |
| 14 | chr2L_14496969_14498135 | nc14F | CAACATGTTTGCAGCCATTC | 488 |
|  |  | nc14R | GTGGGTCTCGCCACCTATTA |  |
| 15 | chrX_3968951_3969506 | nc15F | TTCGGTTGCAGTGGTGATTG | 201 |
|  |  | nc15R | TTCGGTTGCAGTGGTGATTG |  |
| 16 | chr2L_21798044_21799854 | nc16F | TGCTTGAGTGCTTGACCTTG | 458 |
|  |  | nc16R | AAGCACACACACTCACACTG |  |
| 17 | chr3RHet_1825840_1826355 | nc17F | CGCTAGAGAAAAGGCCCAAC | 161 |
|  |  | nc17R | GCGTACCTCAATTGCCTCTG |  |
| 18 | chr3L_18316644_18317098 | nc18F | TGCAAGACCAATCCAAACCG | 254 |
|  |  | nc18R | TGGGAGACAGCACGTAGATT |  |
| 19 | chr3L_18686588_18686941 | nc19F | AGTTCGAAGCCACGGAACTA | 131 |
|  |  | nc19R | CAGGACCTCCTTTTTGTTGC |  |
| 20 | chr2L_8887436_8888049 | nc20F | TCAACATTCAGCGGCAAACA | 374 |
|  |  | nc20R | ATCCGTATCGCTTGGCTACA |  |

# Figure S1


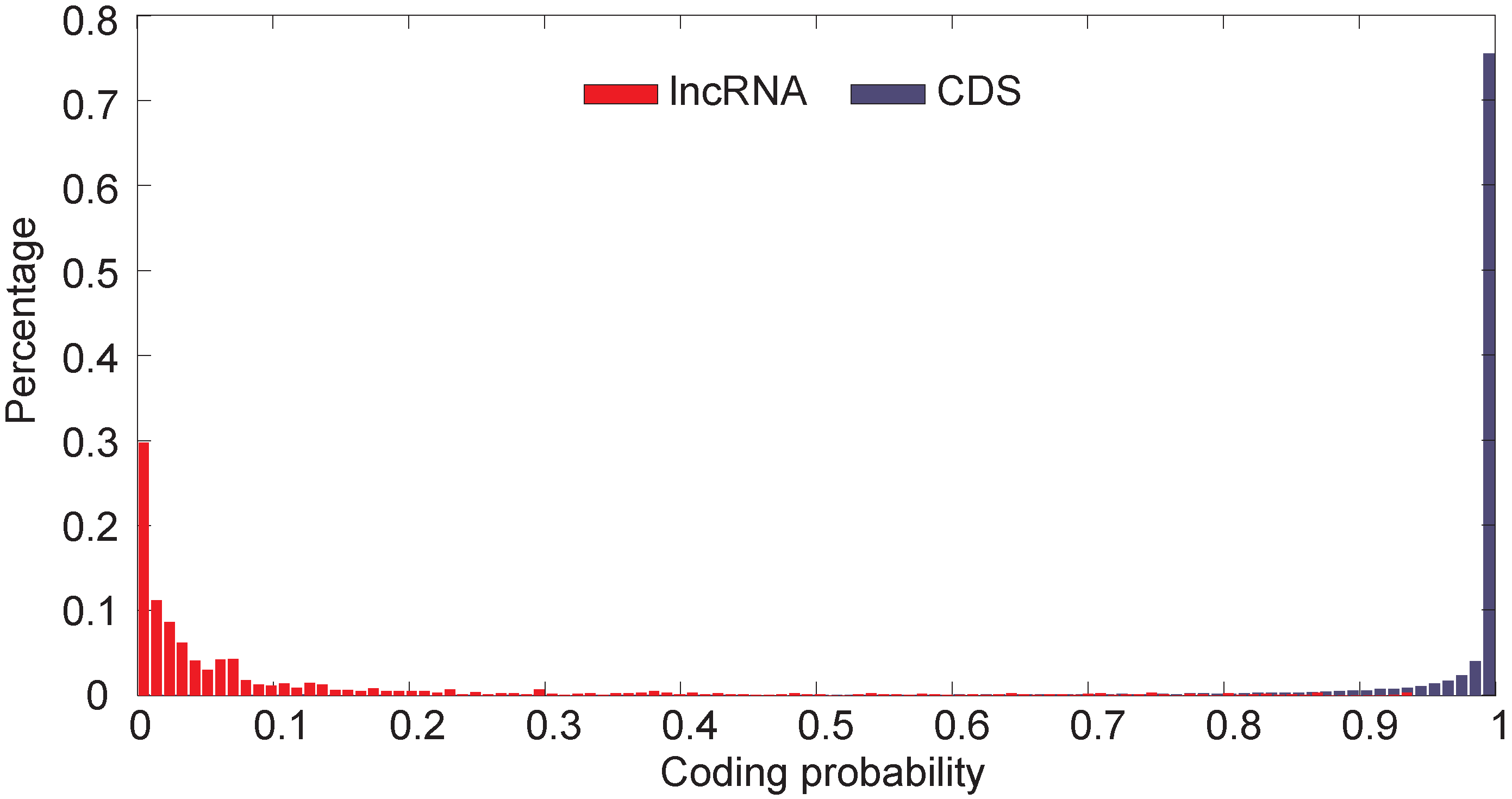


**Supplementary Figure S1.** **Distribution of CPAT scores of the protein-coding transcripts (CDS) and lncRNAs.** The sequences of 33,074 CDS and 976 lncRNAs annotated in this study were analysed for coding probability using CPAT program.

# Figure S2


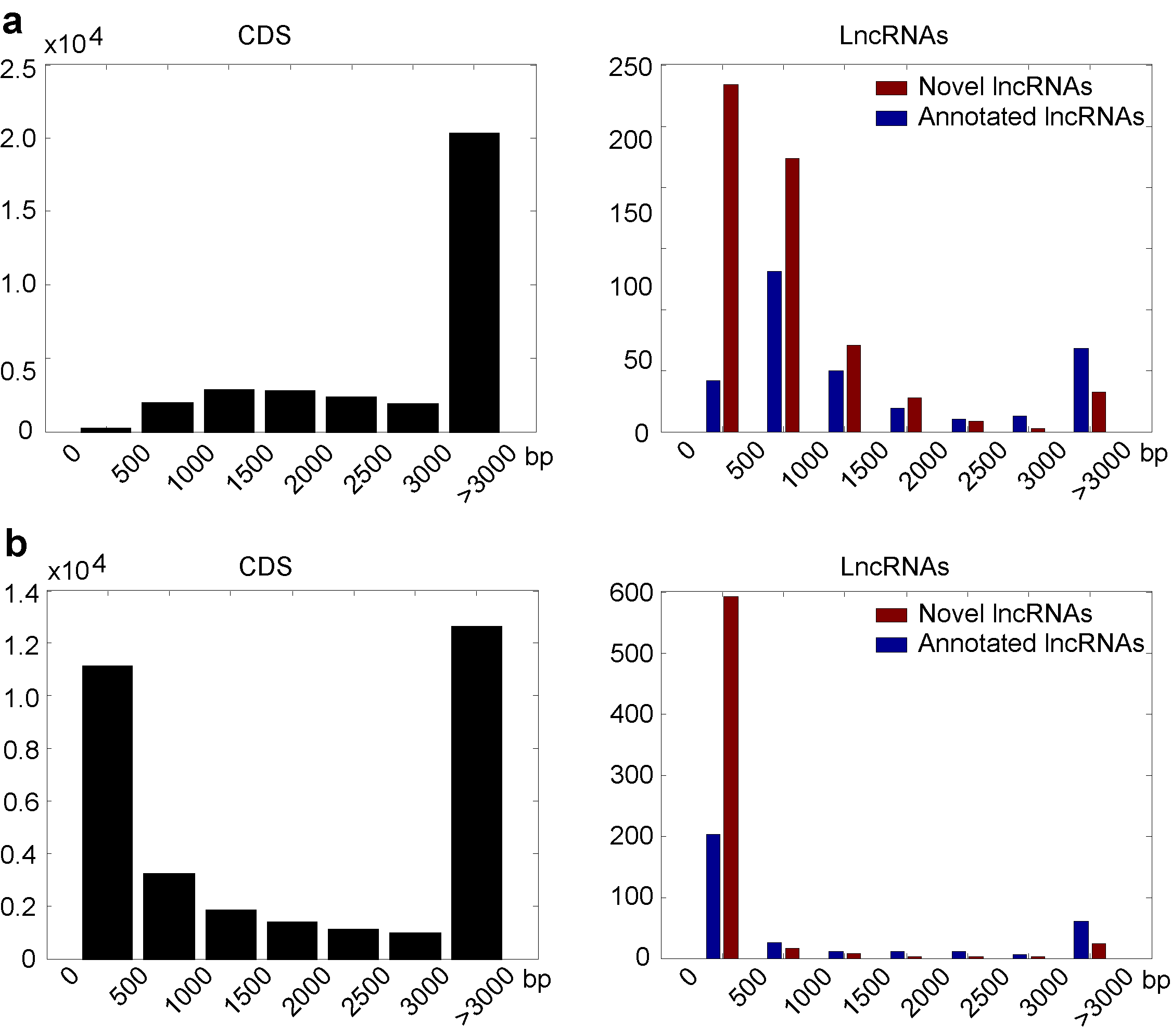


**Supplementary Figure S2.** Size distribution of CDS and lncRNAs in *D. melanogaster***.** (**a**) Total size of CDS and lncRNAs. (**b**) The size of introns of CDS and lncRNAs. The database of *Drosophila* CDS contains 33.074 transcripts. The database of total lncRNAs contains 646 novel lncRNAs and 330 lncRNAs that are annotated in modENCODE and previous publications (see Supplementary Data 1).

# Figure S3


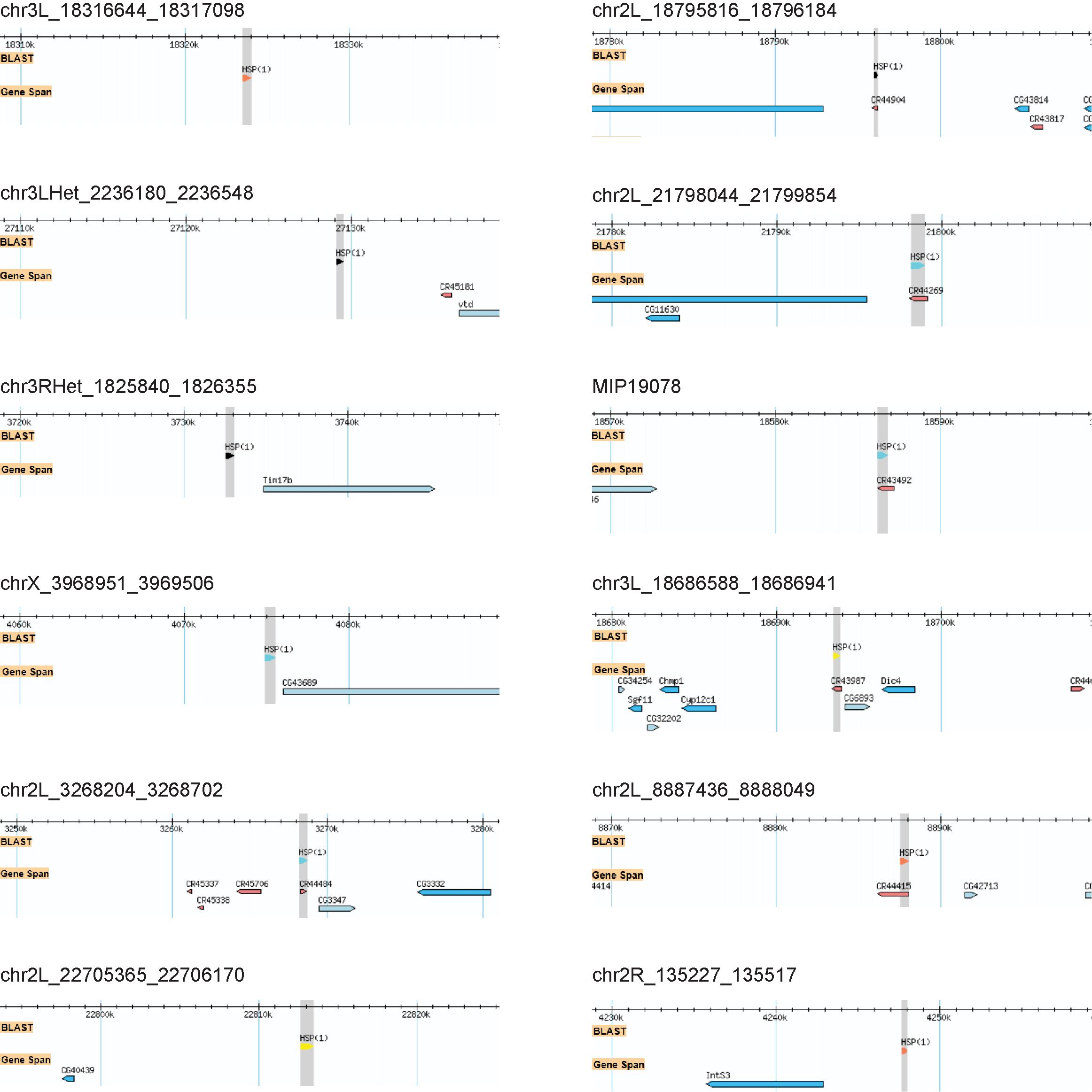


**Supplementary Figure S3.** **Genomic annotation of the 12 *Drosophila* lncRNAs.** Genomic location of the 12 *Drosophila* lncRNAs. The 12 lncRNAs had been validated by RT-PCR and dot hybridization. The chromosome coordinate based on Flybase version FB2014_06 is shown within a range of approximately 30k bp. Hsp(1) indicates the lncRNA after blasting its sequence against the *Drosophila* genome. Other annotated genes in this region are shown below *Gene Span*. See details about the 12 lncRNAs in Table S1 and Supplementary Data 1 online.

# Figure S4


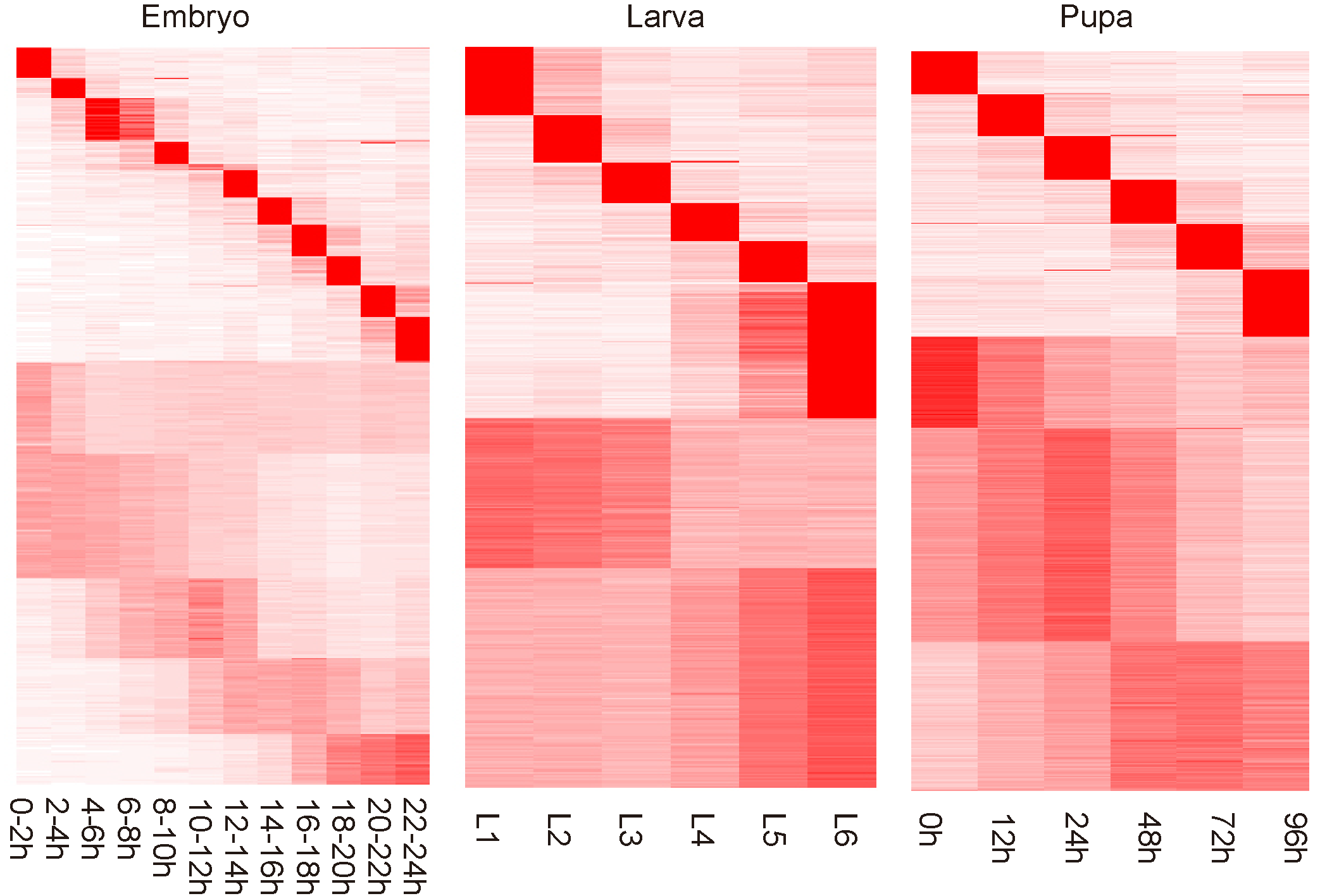


**Supplementary Figure S4.** **Developmental expression of protein-coding genes excluding housekeeping genes in *D. melanogaster*.** The 12 embryonic stages, 6 larval stages and 6 pupal stages (columns) were analyzed. The 32,133 protein-coding RNAs filtered out of 941 housekeeping genes (see Supplementary Data 2) were clustered based on normalized expression values using *k*-means with a city-block distance matrix. *k* = 15, 8, 9 for embryonic, larval and pupal stage, respectively.

# Figure S5


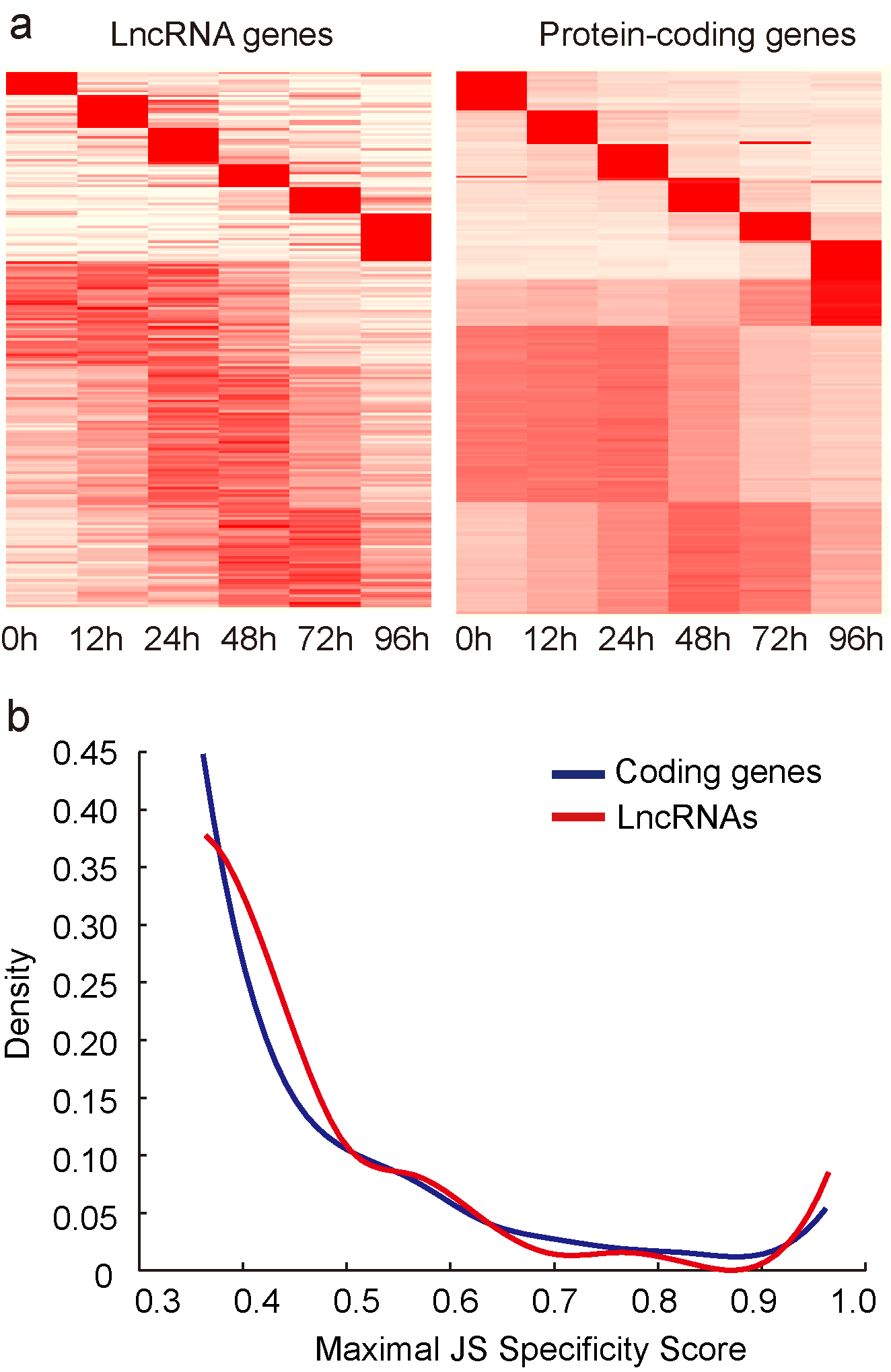


**Supplementary Figure S5.** **Developmental expression of lncRNAs and coding genes at pupal stages in *D. melanogaster*.** The pupal stage is divided into six sub-stages, i.e., prepupae (0 hr), prepupae (12 h), pupae at day 1 (24 h), at day 2 (48 h), at day 3 (73 h), and day 4 (96 h). (**a**) Heatmap of gene expression of lncRNAs and coding gene loci (rows) across 6 pupal stages (columns). The 976 lncRNAs (left) and 33074 protein-coding RNAs (right) were clustered separately based on normalized expression values using k-means (k = 9) with a city-block distance matrix. (**b**) Developmental expression specificity of coding genes and highly expressed lncRNAs. Shown are distributions of Shannon entropy-based temporal specificity scores. The sliding window width is 0.1.
